# SOCIAL RELATIONS AND FRAILTY TRAJECTORY IN LATER ADULTHOOD: EVIDENCE FROM HEALTH AND RETIREMENT STUDY

**DOI:** 10.1093/geroni/igz038.3526

**Published:** 2019-11-08

**Authors:** Ji Hyun Lee

**Affiliations:** Florida State University, Tallahasssee, Florida, United States

## Abstract

Frailty is a state of heightened vulnerability due to the cumulative declines across multiple physiological systems. Growing attention is given to identifying social environmental factors associated with the risk of frailty. It is not yet known how different aspects of social relationships (structure and quality) are linked to frailty, and whether the strength and directions of links may differ by relationship types. Current study aims to 1) to identify sub-populations that follows distinctive trajectory of frailty and 2) to examine the multidimensional social relationship predictors of frailty trajectory. Data came from six waves of the Health and Retirement Study (2006-2016). Sample was older adults aged over 65 (n = 8,892; Mage = 74, SD = 6.96). Frailty index was created using 32 items each wave. Network size, frequency of contact, support, and strain with each relationship type (spouse, children, family, friends) was measured at baseline. Growth mixture model identified three distinctive subpopulations of older adults who share similar frailty trajectory, namely the average, high, and steep increase frailty group. Multinomial logistic regression results showed that frequent contact with friends were associated with lesser frailty. Perceived strain with the spouse, children, and family members all had additive influence on the membership to the higher or steep increase frailty group, compared to the average frailty group. Total network size or perceived support from ties were not significant factors for frailty progression. Interventions can target friendships and stress with kin members as modifiable factors to reduce the risk of frailty progression.

